# Socioeconomic Disparities and Health: Impacts and Pathways

**DOI:** 10.2188/jea.JE20110116

**Published:** 2012-01-05

**Authors:** Naoki Kondo

**Affiliations:** Department of Health Sciences, Interdisciplinary Graduate School of Medicine and Engineering, University of Yamanashi, Yamanashi, Japan

**Keywords:** income inequality, social determinants, economic crisis, socioeconomic status, aged, Japan

## Abstract

Growing socioeconomic disparity is a global concern, as it could affect population health. The author and colleagues have investigated the health impacts of socioeconomic disparities as well as the pathways that underlie those disparities. Our meta-analysis found that a large population has risks of mortality and poor self-rated health that are attributable to income inequality. The study results also suggested the existence of threshold effects (ie, a threshold of income inequality over which the adverse impacts on health increase), period effects (ie, the potential for larger impacts in later years, specifically after the 1990s), and lag effects between income inequality and health outcomes. Our other studies using Japanese national representative survey data and a large-scale cohort study of Japanese older adults (AGES cohort) support the relative deprivation hypothesis, namely, that invidious social comparisons arising from relative deprivation in an unequal society adversely affect health. A study with a natural experiment design found that the socioeconomic gradient in self-rated health might actually have become shallower after the 1997–98 economic crisis in Japan, due to smaller health improvements among middle-class white-collar workers and middle/upper-income workers. In conclusion, income inequality might have adverse impacts on individual health, and psychosocial stress due to relative deprivation may partially explain those impacts. Any study of the effects of macroeconomic fluctuations on health disparities should also consider multiple potential pathways, including expanding income inequality, changes in the labor market, and erosion of social capital. Further studies are needed to attain a better understanding of the social determinants of health in a rapidly changing society.

## INTRODUCTION

**Figure 1.**
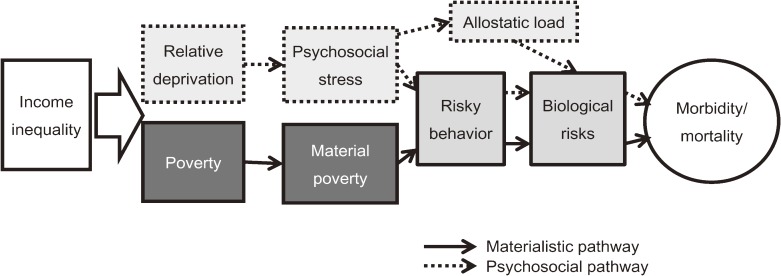
Hypothetical pathways linking income inequality and health

More than three-quarters of the countries belonging to the Organization for Economic Cooperation and Development (OECD) have experienced a growing gap between the rich and poor in the last 2 decades; in addition, growing socioeconomic disparity in the last 3 decades has become a global issue.^1^ Income inequality could damage health via 2 pathways. First, the existence of marked inequality in a society implies that a substantial segment of the population is impoverished, and poverty is bad for health. Second, and more contentiously, income inequality might affect the health of not just the poor, but also society’s more affluent individuals. The so-called spillover effects of inequality have in turn been attributed to psychosocial stress resulting from invidious social comparisons^2^^,^^3^ and the erosion of social cohesion (Figure 1).^4^ With respect to this second pathway, the importance of public health and the burden of income inequality are obviously broader. We have conducted a number of studies that evaluated the contextual impacts of socioeconomic disparities on health and its psychosocial pathways.^2^^,^^3^^,^^5^^,^^6^ We have also investigated whether health disparities increased after the period of economic recession and expanding income inequality in Japan.^7^^,^^8^

## INCOME INEQUALITY HYPOTHESIS: A META-ANALYTIC REVIEW

The income inequality hypothesis implies that income inequality is harmful to all persons, regardless of their absolute income level. This hypothesis has attracted many researchers, and it has been examined in hundreds of studies. Despite the existence of many systematic reviews on this topic, they provide only qualitative evaluations; the debate has thus not reached its conclusion.^4^^,^^9^ To provide quantitative evidence regarding the income inequality hypothesis, my colleagues and I conducted a meta-analysis.^5^

**Figure 2.**
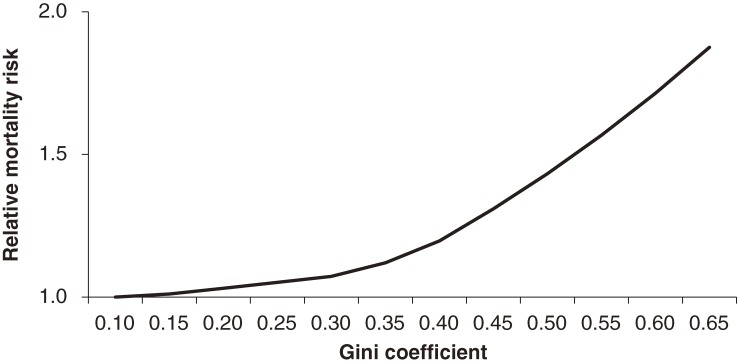
The overall impacts of income inequality (measured by Gini coefficient) on mortality: Results of meta-regression analysis modeling the inequality threshold (original data: Kondo et al, 2009)^5^

In a search of the online data resources PubMed, the ISI Web of Science, and the National Bureau for Economic Research database, we gathered peer-reviewed papers that used multilevel data. Nine cohort studies and 19 cross-sectional studies were eligible. The overall cohort relative risk and cross-sectional odds ratio (OR) (95% confidence interval [CI]) per 0.05-unit increase in Gini coefficient, a measure of income inequality, was 1.08 (1.06–1.10) and 1.04 (1.02–1.06), respectively. Meta-regressions showed stronger associations between income inequality and each of 2 health outcomes among studies with higher Gini values (≥0.3) (Figure 2) that were conducted with post-1990 data, had a longer duration of follow-up (>7 years), and had incorporated time lags between income inequality and outcomes. In contrast, analyses accounting for unmeasured regional characteristics showed a weaker association between income inequality and health. These results suggest a modest adverse effect of income inequality on health, although the population-attributable impact might be larger, given that income inequality could affect a society’s entire population.

The results also support the implications of the threshold effect hypothesis, which posits a threshold of income inequality beyond which adverse impacts on health begin to emerge, as well as lag effects between income inequality and health outcomes, and period effects (ie, potentially larger impacts in later years, specifically after the 1990s).^5^ Our recent study revealed that studies evaluating income inequality in large populations were more likely to show a stronger association between income inequality and health. This could be because a measure of income inequality in a large population might properly capture the levels of social stratification.^10^ However, our study also revealed that threshold, lag, and period effects could be independent of population size.^11^

## EXPLORING PSYCHOSOCIAL PATHWAYS: TESTS OF THE RELATIVE DEPRIVATION HYPOTHESIS

The relative deprivation hypothesis posits that increasing income inequality in a society will heighten an individual’s sense of relative deprivation, resulting in frustration, shame, stress, and maladaptive coping responses (eg, smoking).^12^ The theory of social comparisons underlies this hypothesis.^13^ Empirical support for this hypothesis has been provided recently by studies in Nordic countries^14^^,^^15^ and the United States^16^^,^^17^; however, contrary and less-supportive findings have also been reported,^18^^–^^20^ and there have been no data from Asian countries. Using a large national representative survey dataset from Japan and a large-scale prospective cohort study sample of older adults in Aichi prefecture, Japan, we tested the relative deprivation hypothesis in Japanese populations of adults and older adults.^2^^,^^3^

In the first study, we used data from the 2001 Comprehensive Survey of the Living Conditions of People on Health and Welfare (CSLC), conducted by the Japanese government.^21^ This survey included a probability sample of 22 871 men and 24 243 women aged 25 to 64, from whom information was gathered on demographic variables, household income, occupation or employment status, and self-rated health. Our measure of relative deprivation was the Yitzhaki Index, which calculates deprivation suffered by each individual as a function of the aggregate income shortfall for each person relative to everyone else with higher incomes in that person’s reference group:

$$Yitzhaki\;Index_{i}=\frac{1}{N}\sum_{j}\left(y_{j}-y_{i}\right)\;\forall y_{j}>y_{i},$$

where the amount of relative deprivation for individual *i* is the sum of the income gap between individuals *i* and *j* (*y_j_* − *y_i_*, where *j* has a higher income than *i*) divided by the total number of people in the reference group (*N*). Since we cannot know the reference group of each individual (ie, to whom each person compares him or herself), our approach involves creating alternative definitions for the “reference group”: those with the same occupation, of the same age group, or living in the same geographic area (prefecture), as well as combinations of these. Generalized estimating equations showed that a higher relative deprivation level was associated with worse self-rated health. Even after controlling for absolute income and other sociodemographic factors, the OR (95% CI) for poor self-rated health ranged from 1.09 (1.02–1.16) to 1.18 (1.11–1.26) for men, and from 1.10 (1.04–1.16) to 1.16 (1.09–1.23) for women per 1 million Japanese yen unit increase in the Yitzhaki Index.

In the second study, we used data from the Aichi Gerontological Evaluation Study (AGES), a prospective longitudinal study. In AGES, we conducted a baseline survey in November 2003, with a random sample of functionally independent individuals aged 65 or older residing in 15 municipalities across 3 prefectures in Japan. The sample was restricted to those who were not already receiving public long-term care insurance benefits. Subjects were included only if they reported no limitations in basic activities of daily living, including walking, bathing, and toilet use.^22^ Our study subjects returned the mail-in survey questionnaire, and we analyzed data from 7673 subjects (81%) who had submitted complete information. As a study outcome, we determined the onset of functional disability, based on a new certification for the use of public LTC insurance benefits.^23^ As in our previous approach, we used the Yitzhaki Index, with alternative definitions of “reference group”, namely, others living in the same geographical area (5 municipalities), others in the same age group (65–74 or 75+ years) or of the same sex, others with the same educational attainment (0–9 or 9+ years of education), and combinations of these. A Cox regression analysis showed that after controlling for sociodemographic factors (including absolute income), the hazard ratio (95% CI) of incident physical/cognitive disability per 1 standard deviation increase in relative deprivation ranged from 1.13 (0.99–1.29) to 1.15 (1.01–1.31) in men and from 1.11 (0.94–1.31) to 1.18 (1.00–1.39) in women, depending on the definition of “reference group”. Additional adjustment for lifestyle factors attenuated the hazard ratios to statistical nonsignificance.

In summary, relative deprivation may be a mechanism that underlies the link between income inequality and the health of Japanese adults and older adults. The selection of an unhealthy lifestyle due to stressful psychosocial conditions could in part explain the association between relative deprivation and incident disability.

## ECONOMIC FLUCTUATIONS, EXPANDING INCOME INEQUALITY, AND HEALTH

Japan experienced an economic crisis precipitated by the collapse of the so-called bubble economy. This was followed by more than 2 decades of economic recession, which continues at this writing. Although issues relating to rapid changes in society, the labor market, and expanding socioeconomic disparities are attractive topics of research and debate among many researchers and policy-makers, little is known about the impact of economic fluctuations on health disparities.^7^ Using a natural experiment design, we examined whether socioeconomic-based inequality in self-rated health increased after the 1997–98 economic crisis.^8^^,^^24^ Data on perceived health status, occupation, income, and demographic factors were derived from the 1986, 1989, 1998, and 2001 CSLC. To increase statistical power, data from 1986 and 1989 and those from 1998 and 2001 were pooled. Because we focused on the working-age population, we investigated individuals aged 20 to 60 years. Thus, we used data from a total of 168 801 and 150 016 respondents who completed the income questionnaire for the 1998/2001 and 1986/1989 datasets, respectively. After the economic crisis, the absolute percentages of people reporting poor health declined across all socioeconomic statuses. However, after controlling for confounding factors, the OR (95% CI) for poor self-rated health among middle-class non-manual workers (ie, clerical/sales/service workers) as compared with the highest class of workers (ie, managers/administrators) was 1.02 (0.92–1.14) before the crisis, but increased to 1.14 (1.02–1.29) after the crisis (for temporal change: *P* = 0.02). The association was stronger among men. Adjusted ORs among professional workers and young female homemakers also marginally increased over time. The results showed that self-rated health improved in absolute terms for all occupational groups, even after the economic recession. However, as compared with lower-income people, the self-rated health of people with middle to higher incomes deteriorated after the crisis; unemployed people, however, were twice as likely to report poor health than were the highest class of workers, throughout the period. Unlike commonly observed findings in other countries—namely, the worse the economy, the greater the health inequality^25^^,^^26^—in Japan, the health gradient became shallower after the economic crisis of 1997–98. It was not the health of blue-collar/low-income workers but that of middle-class white-collar workers that declined due to the economic crisis and dramatic changes in social and labor systems.

## CONCLUSION

Society continues to change at a rapid pace in this era of globalization; a better understanding of the social determinants of health is therefore becoming increasingly important. The author and colleagues have investigated social determinants of health, with a special focus on the health impacts of macroeconomic conditions. They found that income inequality and macroeconomic shocks had adverse effects on population health. These effects might be attributable to psychosocial influences that stem from human nature in responding to interpersonal relationships within society. Theories concerning social comparisons and relative deprivation have the potential to explain the pathways that link socioeconomic disparities and health; however, further study is needed to evaluate the health effects of broader social factors. For example, studies on the association between economic fluctuations and society’s health gradient require strong theoretical and empirical underpinnings vis-à-vis pathways and mechanisms. Multiple social aspects could change in times of economic crisis, including labor-market policies, job security, levels of income inequality, and community and workplace social capital; indeed, any of these factors could independently affect health.^27^^,^^28^ Biological mechanisms related to the link between socioeconomic disparities and health also deserve further study.^29^
